# Research on Flight Training Optimization with Instrument Failure Based on Eye Movement Data

**DOI:** 10.3390/jemr18030019

**Published:** 2025-05-23

**Authors:** Jiwen Tai, Yu Qian, Zhili Song, Xiuyi Li, Ziang Qu, Chengzhi Yang

**Affiliations:** 1 School of Flight Technology, Civil Aviation Flight University of China, Guanghan 618307, China; taijw@foxmail.com (J.T.); quziangcafuc@gmail.com (Z.Q.); 13901599769@163.com (C.Y.); 2 Sichuan Provincial Engineering Research Center of Domestic Civil Aircraft Flight and Operation Support, Guanghan 618307, China; 3 Guanghan Campus, Civil Aviation Flight University of China, Guanghan 618307, China; songzhili2222@163.com; 4 School of Civil Aviation Inspector Training, Civil Aviation Flight University of China, Guanghan 618307, China; lixiuyi@cafuc.edu.cn

**Keywords:** eye movement analysis, eye tracking, flight training, instrument failure, flight performance

## Abstract

To improve the quality of flight training in instrument failure scenarios, eye movement data were collected from flight instructors during climbing, descending, and turning flights when the primary attitude direction indicator failed. The performance data of the excellent instructors was selected to produce eye movement tutorials. These tutorials were used to conduct eye movement training for the experimental group of flight trainees. In contrast, the control group received traditional training. The performance and eye movement data of the two groups of flight trainees were then compared and analyzed. The results showed that flight trainees who received eye movement training performed better when facing instrument failure. Specifically, the deviations in the rate of descent, heading during the descent, airspeed during the turn, and slope during the turn were significantly different from those of the control group. Compared to the control group, the experimental group had a significantly lower fixation frequency on the failed instrument during the turn. Additionally, the average glance duration on the failed instrument during the climb and turn was significantly reduced. The study demonstrated the effectiveness of eye movement training in improving the quality of flight training in instrument failure scenarios.

## 1. Introduction

Flight training is a critical aspect of aviation that is directly related to the pilot’s flying skills and flight safety [1]. With the development of aviation technology, flight training on Instrument Flight Rules (IFR) is becoming more and more important [2], because it requires pilots to rely on the airplane’s instruments to control the flight in situations where there is little or no visibility of the external environment [3]. The primary attitude direction indicator provides the pilot with information about the airplane’s attitude relative to the horizon, including pitch and roll angles, to help the pilot be able to understand the airplane’s flight attitude without external visual references [4]. A non-functioning primary attitude direction indicator is one of the emergencies that a pilot may encounter. In real flight, the failure of the primary attitude direction indicator may cause the pilot to lose the accurate perception of the airplane’s attitude, which may lead to serious flight accidents. By training the flight trainees on the failure of the primary attitude direction indicator through simulation training, the trainees can learn how to rely on other flight instruments, such as the altimeter and the airspeed indicator, to maintain the stable flight of the aircraft in a safe environment, so that they can respond quickly and effectively in a real situation. In an emergency such as the failure of the primary attitude direction indicator, the flight trainee relies on other flight instruments and basic flight skills to maintain the ordinary flight of the airplane. At this time, the eye movement characteristics of the flight trainee are critical to achieving safe flight [5]. Flight trainees need to allocate their visual attention appropriately to ensure that they can quickly and accurately acquire and process critical flight information, and to ensure that they can quickly adjust their eye movement behaviors to adapt to the new flight conditions when the primary attitude direction indicator fails. Therefore, it is necessary to conduct an in-depth study on the eye movement characteristics of flight training when the instrument does not work.

The role of visual attention in information processing and task execution has garnered widespread attention from researchers. Souza et al. [6] investigated the role of visual attention in maintaining working memory and found that visual attention plays a primary role in tracking multiple targets. Cirino et al. [7] explored the relationship between visual attention and reading, enhancing the understanding of how attention contributes to reading skills. King et al. [8] discovered evidence that three types of visual working memory representations can simultaneously control attention. Momin et al. [9] examined the relationship between visual attention and mental stress, comprehensively analyzing visual attention under mental stress. The association between cognitive load and visual attention is one of the hot topics of discussion. Liu et al. [10] pointed out that the effect of cognitive load can permeate the early stages of visual processing. Sekicki et al. [11] proposed that a reference gaze would shift visual attention to the cued object, thereby reducing cognitive load. Paladini et al. [12] conducted visual-spatial attention detection experiments under low and high cognitive load conditions. Burtan et al. [13] found through experiments that visual exposure to environmental scenes affects cognitive load.

The application of eye-tracking technology in aviation is a relatively new area of research and a topic of growing interest. The instructor ASsistant SYSTem (iASSYST), which can enhance flight training by providing a record of the pilot’s line of sight, was introduced by Rudi et al. [14] and it was found that iASSYST with eye-tracking capability can support instructors to more accurately identify and categorize the pilot’s errors. Shao et al. [15] examined the relationship between attention span, instrument reading ability, and eye movement patterns, and noted that instrument reading ability is an important factor affecting attention allocation and eye movement patterns during flight and that cognitive resources can be conserved by improving instrument reading ability, thus improving the efficiency of eye movement patterns. Lutnyk et al. [16] explored pilots’ electrodermal activity and gaze behavior in different phases of flight, and found that combining eye-tracking and electrocorticographic activity measurements can more accurately determine pilot status, providing a basis for developing classifiers capable of predicting pilot status. Vlač et al. [17] conducted an in-depth study around how to use eye-tracking tools to improve the selection process of military pilots, and in order to overcome the shortcomings of the single use of traditional eye-tracking metrics that fail to establish a significant correlation with the results of the pilot selection rankings, a network-based representation and three target importance metrics (in-degree, closeness, and betweenness) were introduced to better predict the flight performance and to assist in the military pilot selection process. Behrend et al. [18] analyzed how pilot role (pilot flying vs. pilot monitoring) and rank (captain vs. first officer) affect their decision time and visual attention allocation, pointing out that it is the role assignment rather than the rank that affects the visual gaze on selection-related information. Gao et al. [19] utilized a hidden semi-Markov model (HSMM)-based model to detect the pilot’s visual perceptual state, which can detect and correct the pilot’s perceptual errors promptly, and thus improve the pilot’s situational awareness and reduce the human factor’s impact on aviation safety. Ryffel et al. [20] explored the practical application of eye-tracking technology in general aviation pilots’ loss of control prevention and recovery training (UPRT) evaluations. The use of eye-tracking technology was highly rated by the pilots as it helped to reveal behaviors that needed to be adjusted, facilitated self-awareness, and assisted the instructor in providing personalized feedback. Ke et al. [21] used a virtual reality (VR) flight simulator in conjunction with eye-tracking technology to assess pilots’ flight performance and eye movement patterns and found that participants with flight experience significantly outperformed participants without flight experience in terms of flight performance, showing more structured and efficient eye movement patterns. Babu et al. [22] assessed the cognitive load of pilots in a military aviation environment using eye-tracking technology and noted that eye-tracking technology can provide appropriate warnings to pilots and train military pilots in cognitive load management during operational tasks. Stasi et al. [23] investigated the changes in pilots’ saccadic velocity after long and short simulated flights, and found that the decrease in saccadic velocity was related to the increase in simulated flight time, which suggests that saccadic velocity can be used as an effective biomarker. Lu et al. [24] observed the effects of cognitive load on pilots’ visual search and attention allocation strategies and found that in a high cognitive load task, participants showed shorter mean gaze duration, higher gaze frequency, and lower sweep frequency. Harris et al. [25] examined the virtual reality aviation simulator using eye-tracking technology for its fidelity and effectiveness. Jin et al. [26] conducted eye movement experiments to obtain eye movement features and proposed an air traffic management forgetfulness prediction method based on Convolutional Neural Networks and Long Short-Term Memory Networks (CNN-LSTM). Yang et al. [27] produced an eye movement tutorial for a no-power stall training course and found that the flight performance of the flight trainees receiving eye movement training was significantly improved, which provided an important reference for flight trainees’ attention allocation training. Naeeri et al. [28] focused on whether pilot fatigue increase differed based on their level of specialization (novice vs. expert) and found that experts and novice pilots exhibited different eye movement characteristics. Bruder et al. [29] investigated how experts and novices in the field of aviation differ in their behavior and performance when monitoring automated systems and found that there was no significant difference between experts and novices in their performance in automated fault detection, but that the experts were more efficient in their allocation of attention.

To improve the quality of flight training and ensure flight safety, many scholars have conducted research on flight training methods and their optimization. Dapica et al. [30] proposed an evidence-based training method for training flight instructors, gamified the training process, and verified the feasibility of the training method. Hsu et al. [31] identified and evaluated the risks that existed in flight training, and gave targeted suggestions for further improving the flight training policy, providing an excellent example for flight training risk management in air bases. Arjoni et al. [32] introduced an augmented reality system in formation flight training, and provided a good example for the air force base to improve flight training risk management. Tuhkala et al. [33] pointed out that compared to actual flight, pilots are more likely to judge abnormal situations when training with flight simulators, and it is necessary to include surprise design in flight simulators to improve the closeness of simulated flight training to real flight situations. Xu et al. [34] proposed a mechanism for generating cueing information on the cockpit panel of an aircraft to help flight trainees understand the flight information and to improve the efficiency of flight training. Vitsas et al. [35] applied commercial flight simulator software to flight training, and found that commercial simulators are effective low-cost flight training aids. Wei et al. [36] proposed a new type of flight simulator integrating digital motion cueing algorithms and flight motion platforms, which improves the simulator’s interference resistance and operational efficiency, ensures the safety of training, and enhances training effectiveness. Forrest et al. [37] used deep learning technology to recommend a set of suitable flight events to flight trainees for training, which helped trainees reach the goal of full qualification and accelerated the progress of trainee training. Tatli et al. [38] investigated the impact of meteorological weather on the efficiency of flight training, and found that meteorological weather that can lead to the interruption of flight training is mainly high temperature, heavy fog, and heavy snow. Tong et al. [39] proposed a washout algorithm to reduce the gap between upset prevention and recovery training in a flight simulator and real flight, and improve the fidelity and training effectiveness of simulated flight training. Ahmadi et al. [40] developed an intervention to improve the flight trainee’s gaze method and found that this training method reduced the risk of loss of aircraft control during flight training.

Although many scholars have researched the application of eye movement technology in aviation, few scholars have applied eye movement technology to flight training, especially in emergencies such as when a vital instrument does not work. The role of eye movement technology in optimizing flight training needs to be explored. Based on this, the flight experiment in which the primary attitude direction indicator was set to malfunction was conducted with the SR-20 simulator and Tobii Glasses 3 eye-tracking device. The eye-tracking data for the excellent flight instructors was collected to produce eye-tracking tutorials for the training of the flight trainees in the experimental group. The flight performance and eye-tracking data of those who completed eye movement training were compared with those who were trained traditionally. Statistical methods were used in the comparative analysis to investigate the role of eye movement technology in helping trainees improve their attention allocation and flight performance. The research can provide theoretical references for ensuring flight safety in emergencies, optimizing flight training methods, and improving flight training efficiency.

## 2. Materials and Methods

### 2.1. Subjects

The subjects were flight trainees and instructors from the Guanghan Branch of the Civil Aviation Flight University of China. All flight trainees participating in the experiment need to meet the following conditions: age between 18 and 25, high school education or above, normal or corrected vision and color vision, right-handedness, and no physical defects affecting eye movement data collection. For baseline skills, all theory courses must be passed, with no less than 15 h and no more than 68 h of flight time. This requirement is to ensure that the flight trainees in the experiment have the basic skills to operate the flight simulator and have not experienced training involving instrument failure, to ensure that the baseline skills of the flight trainees are at a relatively consistent level as much as possible. There were six flight instructors, with an average age of 27.6 years (SD = 1.62 years) and an average flight time of 3294 h (SD = 1653.16 h). There were 30 flight trainees, with an average age of 22.37 years (SD = 1.14 years) and an average flight time of 112.8 h (SD = 63.71 h). In arranging the control and experimental groups, stratified random sampling was used to first stratify all participants based on the two key variables of age and flight duration, dividing the age into two age groups, 18–21 and 22–25, and the flight duration into two strata, 15–41 h and 42–68 h. Random assignment to experimental and control groups was then made within each stratum to ensure that the two groups had similar distributions on these key variables. Before starting the experiment, the basic information of the experimental and control groups was statistically analyzed to ensure that there was no significant difference between the two groups of subjects in terms of variables such as age and flight duration (*p* > 0.05), thus ensuring that the results of the experiments were comparable between the two groups.

### 2.2. Equipment

**Figure 1:**
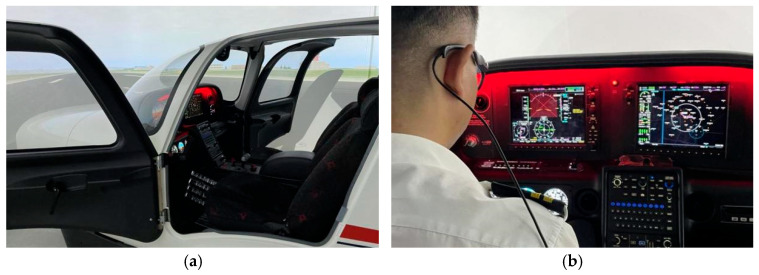
Experimental equipment and scene: (**a**) SR-20 flight simulator and (**b**) scene of the subject wearing the eye tracker for the experiment.

The experimental equipment included an SR-20 flight simulator performing simulated flight tasks and Tobii Glasses 3 collecting eye movement data from the subjects. The SR-20 simulator is a flight simulation training device certified by the Civil Aviation Administration of China. It can simulate each flight stage, and some instruments can be set to not work during the flight. Tobii Glasses 3 is an eyeglass-type eye tracker that can record the eye movement data from the subjects without affecting the flight operation of the subjects, and the sampling rate is 100 Hz. The experimental equipment and experimental scene are shown in Figure 1.

### 2.3. Experimental Procedure

**Figure 2:**
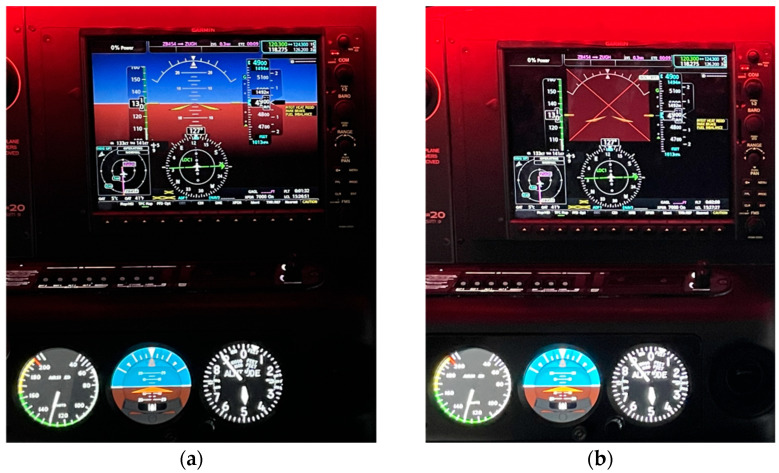
Instrument interfaces when the instrument is normal and when the primary attitude direction indicator is not working: (**a**) when the instrument is normal and (**b**) when the primary attitude direction indicator is not working.

Before the experiment was officially started, the subjects filled in the basic information (flight duration and age, etc.), and wore and calibrated the eye tracker before entering the cockpit. After the beginning of the experiment, the subjects maintained an altitude of 4900 ft, a heading of 127°, and an airspeed of 120 kt, and at this time, all of the instruments were displayed normally. Subsequently, the primary attitude direction indicator of the simulator was set to fail, and other instruments were displayed normally (the standby attitude direction indicator remained normal). After the failure of the primary attitude direction indicator, the subjects needed to climb from 4900 ft to 5900 ft, maintaining an airspeed of 96 kt and a heading of 127°; after climbing to 5900 ft and leveling, they needed to descend from 5900 ft to 4900 ft, keeping the rate of descent of 500 ft/min and heading of 127°; and after lowering to 4900 ft and leveling, they needed to turn left from the heading of 127° to a heading of 0°, maintaining the altitude of 4900 ft and the airspeed of 120 kt, and keeping the slope of 15° during the turning process. After turning to 0°, the task ended. The specific objectives and requirements of the ascending, descending, and turning flight missions are conveyed to the trainees by the instructor. Figure 2 shows the respective instrument interfaces of the SR-20 simulator when the instrument is normal and when the primary attitude direction indicator is not working.

### 2.4. Data Acquisition

#### 2.4.1. Methods of Collecting and Analyzing Eye Movement Data

The study was reviewed and approved by the Ethical Review Committee under ethical review No. 2024 (1). Before the start of the experiment, the principal subject explained to the subjects, in detail, information about the purpose of the study, the process, and how the data would be collected and used, and emphasized that the data collected would be anonymized to protect the rights and privacy of the subjects. The experiment will not have an unintended effect on the subjects’ cognitive workload or stress levels. All subjects voluntarily signed an informed consent form with full understanding.

**Figure 3:**
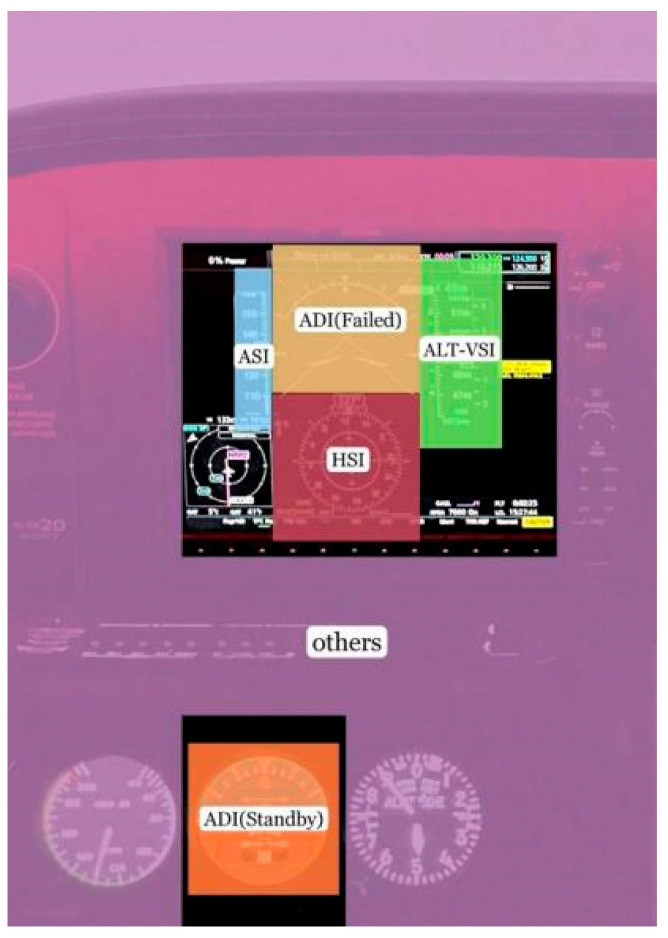
The division of areas of interest.

Throughout the experiment, the original eye movement data from the subjects were collected by Tobii Glasses 3. After the experiment was completed, the collected original eye movement data were imported into Tobii Pro Lab 1.162 software for further processing to obtain the required eye movement index data. There is a built-in I-VT algorithm in Tobii Pro Lab, which can recognize the subjects’ fixation points. In Tobii Pro Lab, the display was divided into multiple areas of interest (AOIs). The division of the AOI is shown in Figure 3. There were six AOIs, including airspeed indicator (ASI), failed primary attitude direction indicator (ADI (Failed)), horizontal situation indicator (HSI), altimeter-vertical speed indicator (ALT-VSI), standby attitude direction indicator (ADI (Standby)), and other areas (others).

#### 2.4.2. Methods of Collecting and Analyzing Flight Data

During the flight task, a high-definition camera was used to record the flight data, such as airspeed, altitude, the rate of descent, heading, and slope, displayed on the instrument interface. The required flight data were obtained by observing the instrument interface in the video frame by frame. The flight process after the primary attitude direction indicator did not work was divided into three stages: climb, descent, and turn. According to the task requirements of the three stages and the suggestions of the instructors, the flight performance indicators and corresponding calculation methods of each stage were proposed, as shown in Table 1. $V_{i}$ represents the airspeed in the ith second of the climb, $H_{i}$ represents the heading in the *i*th second of the climb, ${ROD}_{j}$ represents the rate of descent in the *j*th second of the descent, $H_{j}$ represents the heading in the *j*th second of the descent, $V_{k}$ represents the airspeed in the kth second of the turn, $h_{k}$ represents the altitude in the *k*th second of the turn, and $S_{k}$ represents the slope in the *k*th second of the turn.

### 2.5. Training Methods in Experimental and Control Groups

The traditional flight training model mainly allows flight trainees to observe and imitate the instructor’s maneuvers, combined with the instructor’s verbal instructions for repetitive flight maneuver training. However, since the verbal instruction does not allow the trainee to effectively observe the instructor’s visual focus, the trainee lacks concrete knowledge of the specific way of allocating attention, which affects the quality and efficiency of training. By analyzing the eye movement data of excellent instructors and providing trainees with intuitive eye movement tutorials, it can help trainees understand more clearly the characteristics of attention allocation during flight tasks and improve training effectiveness. A research team from a university had used eye-tracking technology to study the visual scanning pattern and flight decision-making of pilots during a low-visibility approach, and found that there were significant differences in visual patterns and flight decision-making among pilots with different levels of experience, and experienced pilots showed more flexible and adaptive gaze strategies, which provided a scientific basis for the eye movement training of flight trainees based on eye movement data of excellent instructors [41].

The training method used for the subjects in the experimental group was eye movement training. The specific training procedures included static eye movement tutorial learning and dynamic eye movement video tutorial learning. The static eye movement tutorial was mainly to show and explain to the trainees the ratio of the fixation time for each AOI when the excellent instructors were performing flight tasks such as climbing, descending, and turning without the primary attitude direction indicator working, which helped the trainees to understand the allocation of their attention in each AOI when performing different flight tasks. The dynamic eye movement tutorial is a continuity video of the change in the fixation point when the instructor performs the climbing, descending, or turning without the primary attitude direction indicator working. The eye movement video adopts the first viewpoint, and the fixation position is marked with a red circle to show the change in the fixation point by the change in the position of the red circle, which helps the trainees to intuitively learn the dynamics of the attention distribution for the flight in the case of instrument failure. The subjects in the control group were trained traditionally, following the flight training manual for routine training, without providing any data or teaching materials about eye movement.

The data used to make the eye movement training tutorials was obtained from selected excellent instructors. Excellent instructors were required to meet the following flight performance criteria: ascent heading deviation of no more than 2.5°, ascent airspeed deviation of no more than 5 kt, descent heading deviation of no more than 2.5°, descent rate deviation of no more than 200 ft/min, turn airspeed deviation of no more than 10 kt, turn altitude deviation of no more than 50 ft, and turn slope deviation of no more than 5°. This performance standard was determined through discussion with expert pilots.

**Figure 4:**
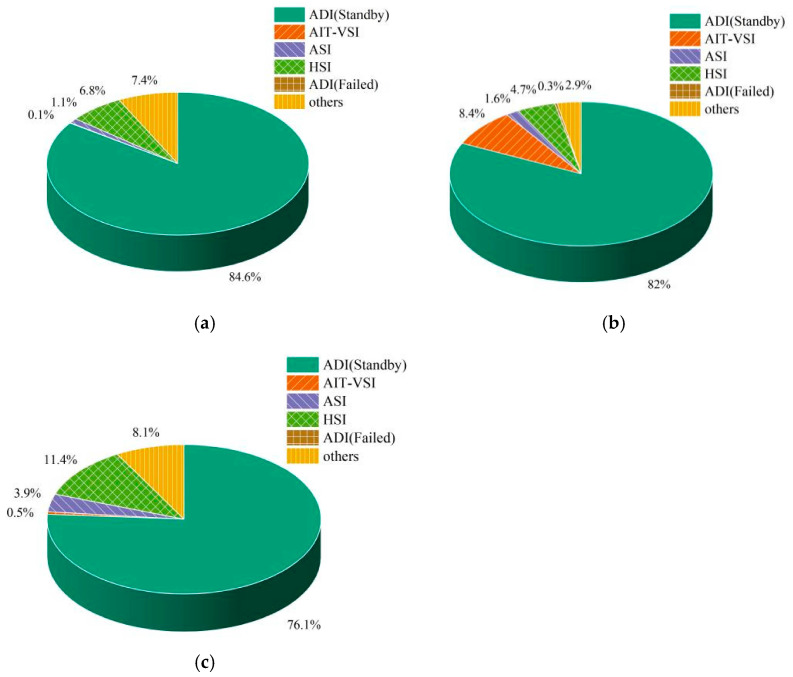
Percentage of fixation time of excellent instructors in each AOI when the primary attitude direction indicator was not working: (**a**) climb, (**b**) descent, and (**c**) turn.

The proportion of the fixation time of excellent instructors in each AOI is shown in Figure 4. It can be found that when the primary attitude direction indicator was not working, the AOI of ADI (Standby) was the area with the largest proportion of fixation time in the climb, descent, and turn. The AOI of ADI (Failed) was the area with the smallest proportion of fixation time in each stage. This is because after the primary attitude direction indicator fails, it can no longer provide related flight information. It is necessary to observe the ADI (Standby) to replace the ADI (Failed), to continue obtaining attitude information such as flight slope and pitch angle.

The tutorial video of the first perspective of the excellent flight instructors was divided into three stages: climb, descent, and turn. The tutorial video for each stage was selected from the flight instructor with the best flight performance at this stage. The red circle was used to indicate the instructor’s fixation position at each moment, which provided an intuitive attention allocation reference for the experimental group to learn to complete the flight task without the primary attitude direction indicator working.

The control group with traditional training according to the flight training manual and was not provided with eye movement-related data.

## 3. Results

### 3.1. Differences Between Groups in the Climbing Flight When the Primary Attitude Direction Indicator Failed

#### 3.1.1. Differences in Flight Performance Between Groups in the Climbing Flight

**Figure 5:**
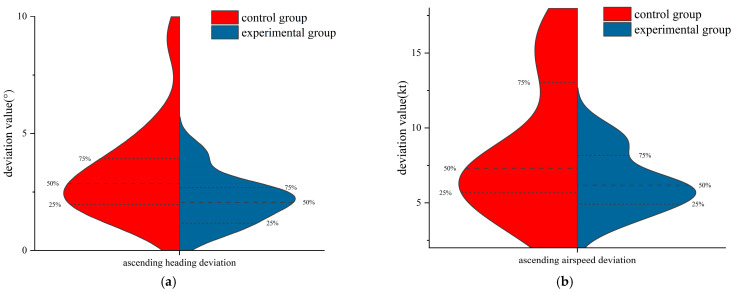
The violin plot of flight performance of the two groups of subjects in the climbing flight: (**a**) ascending heading deviation and (**b**) ascending airspeed deviation.

In the climbing flight, to study the differences in flight performance between the two groups of flight trainees trained in different methods, the normality test was performed on the airspeed deviation and heading deviation of the two groups. The results showed that the data generally obeyed the normal distribution. The independent samples *t*-test was used to examine the differences. The results of the independent samples *t*-test are shown in Table 2. Figure 5 is the violin pilot comparing the flight performance of the two groups. There was no significant difference between the two groups in the heading deviation and the airspeed deviation (*p* > 0.05). However, the mean value of the airspeed deviation and the mean value of the heading deviation in the experimental group were less than those in the control group. Additionally, it also can be seen from Figure 5 that the 75%, 50%, and 25% digits of the heading deviation and the airspeed deviation in the experimental group were less than those in the control group, indicating that although the flight performance of the experimental group was not significantly different from that of the control group, the ability of the trainees trained by eye movement tutorials to maintain airspeed and flight heading during the climb with the primary attitude direction indicator not working was improved to a certain extent.

#### 3.1.2. Differences in Eye Movement Indicators Between Groups in the Climbing Flight

In the climbing flight, to study the differences in eye movement indicators between the two groups of flight trainees trained in different methods, the normality test was performed on the eye movement indicators of the two groups. The results showed that the data generally obeyed the normal distribution. The independent samples *t*-test was used to examine the differences.

**Figure 6:**
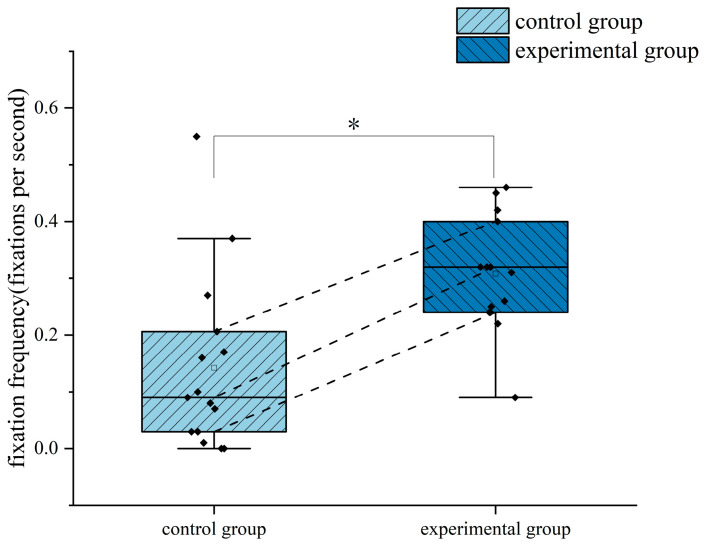
Difference in the fixation frequency in the ASI area between the two groups of subjects in the climb phase. *: *p* < 0.05.

In terms of fixation frequency, the results of the independent samples *t*-test showed that there was a significant difference between the two groups of trainees in terms of fixation frequency in the ASI area (*p* = 0.002 < 0.05), with a Cohen’s *d* value of 1.278 and a 95% confidence interval of (−0.263, −0.068). The frequency of fixation in the ASI area was significantly higher for the trainees trained by the eye movement tutorial than for the trainees trained conventionally, indicating that the trainees who completed the eye movement training paid more attention to the airspeed indicator and were able to grasp the airspeed changes during the climb to ensure the required ascent airspeed. The difference in the fixation frequency of the two groups of trainees in the ASI area during the climbing flight is shown in Figure 6. The star symbol indicates that the result is statistically significant. The diamond symbol represents the numerical value of the sample. The square symbol represents the average value.

**Figure 7:**
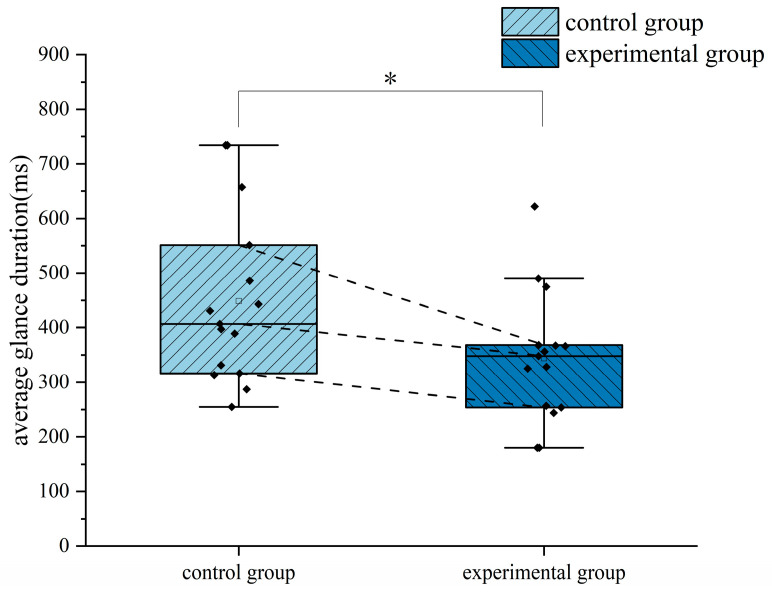
Difference in the average glance duration in the ADI (Failed) area between the two groups of subjects in the climbing flight. *: *p* < 0.05.

In terms of the average glance duration, the independent samples *t*-test results showed that there was a significant difference in the average glance duration between the two groups in the ADI (Failed) area (*p* = 0.048 < 0.05), with a Cohen’s *d* value of 0.756 and a 95% confidence interval of (1.102, 208.365). The average glance duration in the ADI (Failed) area of the trainees trained by eye movement tutorials was significantly shorter than that of the trainees trained traditionally, indicating that the trainees in the experimental group allocated their attention more effectively, and could quickly scan the failed instrument interface and ignore it, focusing on the instrument that was still in operation, rather than wasting more time on the failed instrument. The difference in the average glance duration in the ADI (Failed) area between the two groups during the climbing flight is shown in Figure 7. The star symbol indicates that the result is statistically significant. The diamond symbol represents the numerical value of the sample. The square symbol represents the average value.

### 3.2. Differences Between Groups in the Descending Flight When the Primary Attitude Direction Indicator Failed

#### 3.2.1. Differences in Flight Performance Between Groups in the Descending Flight

**Figure 8:**
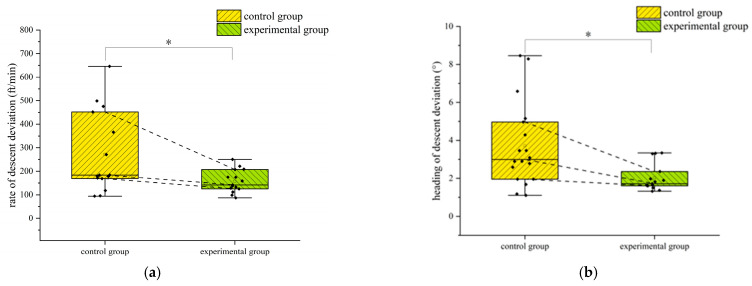
Differences in flight performance between the two groups of subjects in the descending flight: (**a**) rate of descent deviation and (**b**) heading of descent deviation. *: *p* < 0.05.

In the descending flight, to study the differences in flight performance between the two groups of flight trainees trained in different methods, the normality test was performed on the deviations in the heading and rate of descent of the two groups. The results showed that the data generally obeyed the normal distribution. The independent samples *t*-test was used to examine the differences. The results of the independent samples *t*-test are shown in Table 3. The differences in the flight performance between the two groups of trainees are shown in Figure 8. The star symbol indicates that the result is statistically significant. The diamond symbol represents the numerical value of the sample. The square symbol represents the average value. The results of the independent samples *t*-test showed significant differences between the two groups in the deviations in the rate of descent and heading (*p* < 0.05). The deviations in the rate of descent and heading of the trainees trained by eye movement tutorials were significantly less than those of the trainees trained traditionally when the primary attitude direction indicator was not working, indicating that the ability of the trainees who completed eye movement training to maintain the rate of descent and stabilize the flight heading in the descending flight was significantly enhanced.

#### 3.2.2. Differences in Eye Movement Indicators Between Groups in the Descending Flight

In the descending flight, to study the differences in eye movement indicators between the two groups of flight trainees trained in different methods, the normality test was performed on the eye movement indicators of the two groups. The results showed that the data generally obeyed the normal distribution. The independent samples *t*-test was used to examine the differences.

**Figure 9:**
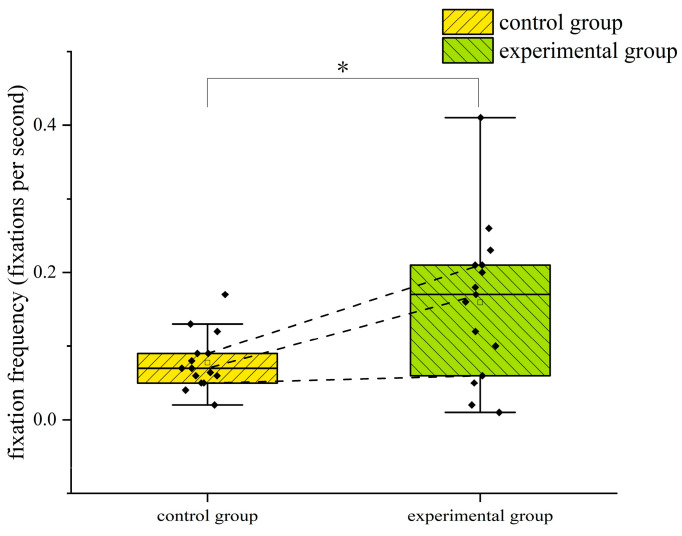
Difference in the fixation frequency in the ASI area between the two groups of subjects during the descending flight. *: *p* < 0.05.

In terms of fixation frequency, the independent samples *t*-test results showed that there was a significant difference between the two groups of trainees in the ASI area (*p* = 0.009 < 0.05), with a Cohen’s *d* value of 1.063 and a 95% confidence interval of (−0.145, −0.023). The trainees who completed the eye movement training had a significantly higher fixation frequency in the ASI area than those who had the traditional training, which indicated that the trainees in the experimental group were able to pay more attention to the change in the airspeed in the process of descending while maintaining a fixed descent rate. This may be because airspeed tends to increase during descent, and excessive airspeed is not conducive to the precise control of the descent rate. Trainees who completed the eye movement training were able to focus on the airspeed indicator while paying attention to the rate of descent, whereas those who did not receive the eye movement training tended to ignore the attention to airspeed during descent. The difference in fixation frequency in the ASI area between the two groups during the descending flight is shown in Figure 9. The star symbol indicates that the result is statistically significant. The diamond symbol represents the numerical value of the sample. The square symbol represents the average value.

### 3.3. Differences Between Groups in the Turning Flight When the Primary Attitude Direction Indicator Failed

#### 3.3.1. Differences in Flight Performance Between Groups in the Turning Flight

**Figure 10:**
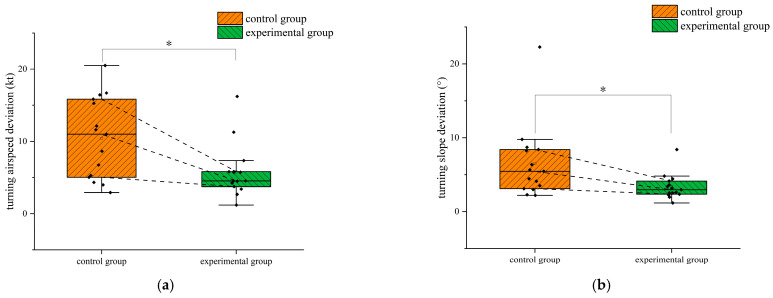
Differences in turning airspeed deviation and slope deviation between the two groups of subjects in the turning flight: (**a**) turning airspeed deviation and (**b**) turning slope deviation. *: *p* < 0.05.

**Figure 11:**
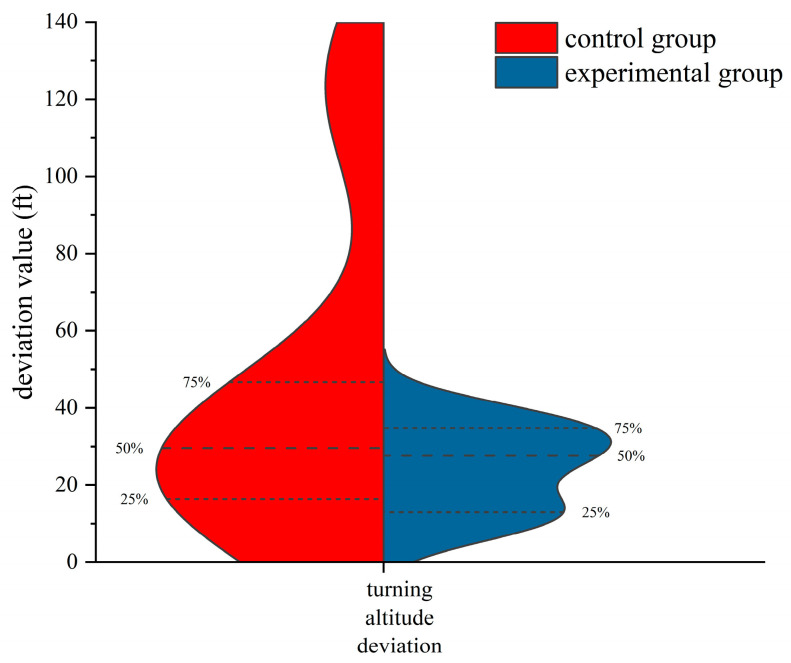
The violin plot of the turning altitude deviation of the two groups of subjects during the turning flight.

In the turning flight, to study the differences in flight performance between the two groups of flight trainees trained in different methods, the normality test was performed on the airspeed deviation, altitude deviation, and slope deviation of the two groups. The results showed that the data generally obeyed the normal distribution. The independent samples *t*-test was used to examine the differences. The results of the independent samples *t*-test are shown in Table 4. The differences in turning airspeed deviation and slope deviation between the two groups of trainees are shown in Figure 10. The star symbol indicates that the result is statistically significant. The diamond symbol represents the numerical value of the sample. The square symbol represents the average value. Figure 11 is the violin pilot comparing the turning altitude deviation of the two groups. The results of the independent samples *t*-test showed significant differences between the two groups in the deviations in the turning airspeed and slope (*p* < 0.05). The deviations in the airspeed and slope of the trainees who completed eye movement training were significantly less than those of the trainees trained traditionally when the primary attitude direction indicator did not work, indicating that the trainees in the experimental group could better maintain the airspeed and slope of the turn when the primary attitude direction indicator failed. Although there was no significant difference in the deviation in turning altitude between the two groups, the average altitude turning deviation in the experimental group was less than that in the control group. Additionally, through Figure 11, it can be seen that the 75%, 50%, and 25% digits of the deviation in turning altitude in the experimental group were less than those in the control group, indicating that the ability of trainees with eye movement training to maintain turning altitude was improved to a certain extent.

#### 3.3.2. Differences in Eye Movement Indicators Between Groups in the Turning Flight

In the turning flight, to study the differences in eye movement indicators between the two groups of flight trainees trained in different methods, the normality test was performed on the eye movement indicators of the two groups. The results showed that the data generally obeyed the normal distribution. The independent samples *t*-test was used to examine the differences.

**Figure 12:**
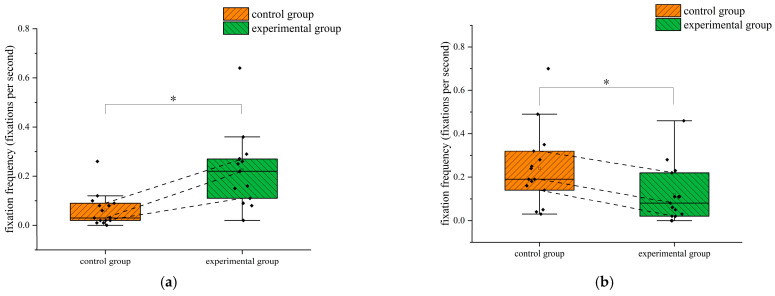
Differences in the fixation frequency between the two groups of subjects in the ASI and ADI (Failed) areas during the turning flight: (**a**) ASI and (**b**) ADI (Failed). *: *p* < 0.05.

In terms of fixation frequency, the independent samples *t*-test results indicated that there was a significant difference between the two groups in the ASI area (*p* = 0.001 < 0.05), with a Cohen’s *d* value of 1.424 and a 95% confidence interval of (−0.250, −0.076), and the ADI (Failed) area (*p* = 0.04 < 0.05), with a Cohen’s *d* value of 0.786 and a 95% confidence interval of (0.005, 0.238). Trainees who received eye movement training had significantly higher fixation frequency in the ASI area than those who received conventional training, while fixation frequency in the ADI (Failed) area was significantly lower than those who received conventional training. This suggests that trainees in the experimental group could pay more attention to airspeed changes during flight turns to maintain the required turn airspeed, pay less attention to the failed instrument, and focus their attention on the effective instrument. The differences in fixation frequency between the two groups in the ASI and ADI (Failed) areas during the turning flight are shown in Figure 12. The star symbol indicates that the result is statistically significant. The diamond symbol represents the numerical value of the sample. The square symbol represents the average value.

**Figure 13:**
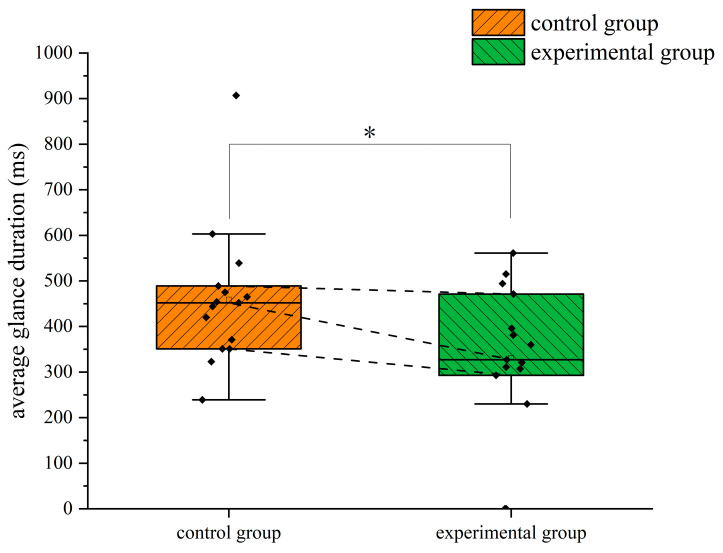
Difference in the average glance duration in the ADI (Failed) area between the two groups of subjects during the turning flight. *: *p* < 0.05.

In terms of the average glance duration, the independent samples *t*-test results show that there was a significant difference in the average glance duration in ADI (Failed) between the two groups (*p* = 0.035 < 0.05), with a Cohen’s *d* value of 0.807 and a 95% confidence interval of (9.370, 246.097). The average glance duration in ADI (Failed) of the trainees trained by eye movement is significantly shorter than that of the trainees trained traditionally, which shows that the trainees in the experimental group can identify the failed instrument more quickly during turning and turn their attention to other instruments. Combined with the fixation frequency, it can be seen that eye movement training can help trainees avoid the interference of the failed instrument on their attention and adopt more effective visual search strategies. The difference in the average glance duration between the two groups of trainees in the ADI (Failed) area during the turning flight is shown in Figure 13. The star symbol indicates that the result is statistically significant. The diamond symbol represents the numerical value of the sample. The square symbol represents the average value.

## 4. Discussion

In flight training with instrument failure, eye movement training helped flight trainees significantly reduce the deviations in the rate of descent, heading of descent, airspeed of turns, and slope of turns. The deviations in the heading and airspeed in the climb and the altitude deviation in the turn were also decreased. In terms of attention distribution, when climbing with instrument failure, compared with the control group trained traditionally, the fixation frequency of the airspeed indicator of the experimental group with eye movement training was significantly higher, and the average glance duration of the failed instrument was significantly shorter. When descending, the flight trainees with eye movement training had a significant increase in the fixation frequency of the airspeed indicator. When turning, there was a significant increase in the fixation frequency of the airspeed indicator and a significant decrease in the fixation frequency and average glance duration of the failed instrument of the flying trainees with eye movement training. Eye movement training helps flight trainees optimize attention distribution, weaken attention to the failed instrument, and focus more on instruments that are still working, to obtain effective flight information, reasonably control the aircraft to complete flight tasks, and achieve ideal flight results.

Chan, A.S. et al. [42] used eye-tracking technology to train children with attention-deficit/hyperactivity disorder (ADHD) and autism and found that eye-tracking technology can improve their visual information memory ability and flexible thinking ability. Yang, J., et al. [27] found that pilots who received eye movement training can better control the stalled aircraft, lose less height and speed, and take less time to return the aircraft from the unstable state to the normal stable state, which is consistent with the conclusion of this study. Eye movement training is an effective way to help pilots improve flight performance. Compared with previous studies, this study applied eye movement training to the specific scenario of instrument failure, designed a three-stage task of climbing, descending, and turning, and comprehensively analyzed the effect of eye movement training on improving flight performance in the scenario of instrument failure.

**(1)**  **Real-world application considerations**

To apply eye movement research to real-world flight training, flight training organizations should introduce eye-tracking technology. This involves training flight instructors in the relevant techniques to ensure their proficiency in using eye-tracking equipment and interpreting the data. High-quality eye-tracking equipment should be installed in flight simulators or actual aircraft to record eye movement data. Corresponding eye movement training courses should be developed for different flight tasks and training disciplines, covering key skills such as optimizing attention allocation and quickly adjusting fixation points. Training can be conducted in phases, with increasing difficulty from basic simulator training to real flight applications.

During the implementation process, a combination of simulator and real flight training should be used to allow trainees to practice in different environments and adapt to various flight conditions. Eye movement data and flight performance data should be regularly collected and analyzed to provide personalized feedback to each trainee, helping them improve their attention allocation and flight operations. The training program should be continuously improved by incorporating advanced technologies such as virtual reality to offer a more immersive training experience. The effectiveness of the training should be regularly assessed, and the content and methods should be adjusted based on feedback. Successful experiences should be extended to more flight training organizations, and collaboration with aviation authorities should be pursued to promote the inclusion of eye movement training in industry standards.

**(2)**  **Limitations of simulation environment and its countermeasures**

Although the simulation environment has many advantages in flight training, such as high safety and controllable cost, it also has certain limitations. The simulation environment cannot completely replicate the sensory experience in real flight, which may lead to bias in the pilot’s judgment of spatial position in real flight [43]. The difference in psychological stress is also an important issue [44]. In a simulation environment, pilots usually do not feel the sense of urgency that they do in a real flight because errors in the simulator do not have practical consequences. This psychological difference may affect the speed and accuracy of pilots’ decision-making in emergencies [45]. In addition, the simulation environment is usually less complex than real flight. For example, weather conditions and mechanical failures in simulators are often pre-determined and lack the unpredictability and dynamic changes found in real flight [46]. These limitations may make it difficult for pilots to adapt quickly when encountering unexpected situations in real flight.

In order to overcome these limitations, the following steps can be taken in real-world applications in the future. First, virtual reality and augmented reality technologies can be combined to enhance the realism of the simulation environment. By introducing more realistic visual and auditory effects, as well as motion cues in simulation flight, the immersion and perception of the pilot can be improved. Second, the complexity of the simulation environment can be increased by introducing dynamic and unpredictable training scenarios. For example, randomly occurring weather changes and mechanical failures can be designed so that pilots face more challenges during training. In addition, simulation training can be combined with real flight training in a gradual transition. For example, basic training is conducted in the simulator first, and then applied and consolidated in real flight. In this way, pilots can gradually adapt to complex and unpredictable situations in a real environment.

**(3)**  **Potential disadvantages of eye movement training and its countermeasures**

Eye movement training showed many advantages in this study, but there are some potential disadvantages. The prolonged use of eye-tracking devices may trigger visual fatigue or mental fatigue, especially during high-intensity flight training. The eye-tracking device itself may distract the trainee’s attention. The trainee may focus too much on the use or feedback of the device and neglect the actual operation of the flight instruments, which may lead to safety hazards in real flight. In addition, eye movement training is costly and requires a significant investment of resources for equipment acquisition, maintenance, and training.

To overcome these shortcomings, the following measures can be taken in real-world applications in the future:

- Rationalize the training time, avoid prolonged and continuous use, and reduce the visual and mental burden of the trainees.
- Develop lighter and more comfortable eye-tracking devices, and avoid or minimize as much as possible the interference of the eye-tracking devices themselves on flight operations and attention.
- For some flight training organizations with limited budgets, eye-tracking equipment rental can be considered. The leasing model can reduce the initial investment cost, and at the same time, can flexibly adjust the leasing period according to the actual needs, avoiding the waste of resources caused by idle equipment.

To sum up, eye movement training in this study achieved significant results in improving flight performance and attention allocation in a simulation environment, but it is crucial to consider its practical applications and limitations comprehensively. By addressing these aspects, future research and practical applications can better utilize the benefits of eye movement training to enhance aviation safety and training quality.

## 5. Conclusions

Focusing on instrument failure scenarios in flight training, this study aimed to optimize flight trainees’ attention allocation in emergencies and enhance the quality of flight training through eye movement training. Eye movement data from flight instructors when they encountered primary attitude direction indicator failure was collected, and the eye movement tutorials were created accordingly. Flight trainees in the experimental group received training based on the eye movement tutorial, while the control group received traditional training.

Comparing the two groups’ flight performance and eye movement data during instrument failure revealed that eye movement training significantly reduced deviations in key flight parameters such as the rate of descent, heading of descent, airspeed of turns, and slope of turns. It also optimized attention distribution, decreasing fixation frequency and glance duration on the failed instrument and increasing focus on functional instruments.

However, this research was conducted in a simulation environment, which, despite its advantages, cannot fully replicate the sensory experiences, psychological pressures, and complexities of real flight conditions. This limitation may affect the direct applicability to actual flight training. Future studies should explore real flight environments or integrate advanced technologies like virtual reality to enhance the external validity of research results. Additionally, extending eye movement training to broader flight training scenarios, especially emergency situations, could further improve emergency response capabilities and overall flight safety. Investigating the long-term effects of eye movement training is also essential for developing comprehensive flight training programs.

In summary, this study offers valuable insights into optimizing flight trainees’ attention allocation through eye movement training, demonstrating its potential to improve flight performance and emergency response capabilities. Future research and practical applications should consider the limitations of simulation environments and explore ways to maximize the benefits of eye movement training in enhancing aviation safety and training quality.

## Figures and Tables

**Table 1:** Flight performance indicators for each stage when the primary attitude direction indicator was not working.

**Stage**	**Time (s)**	**Flight Performance Indicator (Unit)**	**Calculation Formula**
Climb	*m*	the deviation in airspeed (kt)	$\frac{1}{m}\sum_{i=1}^{m}|V_{i}-96|$
		the deviation in heading (°)	$\frac{1}{m}\sum_{i=1}^{m}|H_{i}-127|$
Descent	*n*	the deviation in rate of descent (ft/min)	$\frac{1}{n}\sum_{j=1}^{n}|{ROD}_{j}-500|$
		the deviation in heading (°)	$\frac{1}{n}\sum_{j=1}^{n}|H_{j}-127|$
Turn	*p*	the deviation in airspeed (kt)	$\frac{1}{p}\sum_{k=1}^{p}|V_{k}-120|$
		the deviation in altitude (ft)	$\frac{1}{p}\sum_{k=1}^{p}|h_{k}-4900|$
		the deviation in slope (°)	$\frac{1}{p}\sum_{k=1}^{p}|S_{k}-15|$

**Table 2:** Independent samples *t*-test results of flight performance of two groups of subjects in the climbing flight.

Flight Performance	Group	Mean Value	*p*-Value	95% Confidence Interval
ascending heading deviation	control group	3.2997	0.065	(−0.071, 2.274)
	experimental group	2.1981		
ascending airspeed deviation	control group	8.4766	0.113	(−0.551, 4.772)
	experimental group	6.3661		

**Table 3:** Independent samples *t*-test results of flight performance of two groups of subjects in the descending flight.

Flight Performance	Group	Mean Value	*p*-Value	95% Confidence Interval	Cohen’s *d*
rate of descent deviation	control group	272.2129	0.024	(17.451, 212.613)	0.912
	experimental group	157.1812			
heading of descent deviation	control group	3.8989	0.010	(0.518, 3.226)	1.068
	experimental group	2.0270			

**Table 4:** Independent samples *t*-test results of flight performance of two groups of subjects in the turning flight.

Flight Performance	Group	Mean Value	*p*-Value	95% Confidence Interval	Cohen’s *d*
turning airspeed deviation	control group	10.4309	0.013	(1.070, 8.216)	0.979
	experimental group	5.7881			
turning altitude deviation	control group	44.3888	0.093	(−3.767, 44.044)	/
	experimental group	24.2506			
turning slope deviation	control group	6.5034	0.034	(0.266, 6.030)	0.841
	experimental group	3.3554			

## Data Availability

The original contributions presented in the study are included in the article; further inquiries can be directed to the corresponding author.
